# Determining the Genetic Characteristics of Resistance and Virulence of the “Epidermidis Cluster Group” Through Pan-Genome Analysis

**DOI:** 10.3389/fcimb.2020.00274

**Published:** 2020-06-12

**Authors:** Zhewei Sun, Danying Zhou, Xueya Zhang, Qiaoling Li, Hailong Lin, Wei Lu, Hongmao Liu, Junwan Lu, Xi Lin, Kewei Li, Teng Xu, Qiyu Bao, Hailin Zhang

**Affiliations:** ^1^Key Laboratory of Medical Genetics of Zhejiang Province, Key Laboratory of Laboratory Medicine, Ministry of Education, School of Laboratory Medicine and Life Sciences, Wenzhou Medical University, Wenzhou, China; ^2^The Second Affiliated Hospital and Yuying Children's Hospital, Wenzhou Medical University, Wenzhou, China; ^3^Institute of Biomedical Informatics, Wenzhou Medical University, Wenzhou, China; ^4^Institute of Translational Medicine, Baotou Central Hospital, Baotou, China

**Keywords:** Epidermidis Cluster Group, *bla* operon, resistance, *isd* locus, virulence, pan-genome

## Abstract

*Staphylococcus caprae, Staphylococcus capitis*, and *Staphylococcus epidermidis* belong to the “Epidermidis Cluster Group” (ECG) and are generally opportunistic pathogens. In this work, whole genome sequencing, molecular cloning and pan-genome analysis were performed to investigate the genetic characteristics of the resistance, virulence and genome structures of 69 ECG strains, including a clinical isolate (*S. caprae* SY333) obtained in this work. Two resistance genes (*blaZ* and *aadD2*) encoded on the plasmids pSY333-41 and pSY333-45 of *S. caprae* SY333 were confirmed to be functional. The *bla* region in ECG exhibited three distinct structures, and these chromosome- and plasmid-encoded *bla* operons seemed to follow two different evolutionary paths. Pan-genome analysis revealed their pan-genomes tend to be “open.” For the virulence-related factors, the genes involved in primary attachment were observed almost exclusively in *S. epidermidis*, while the genes associated with intercellular aggregation were observed more frequently in *S. caprae* and *S. capitis*. The type VII secretion system was present in all strains of *S. caprae* and some of *S. epidermidis* but not in *S. capitis*. Moreover, the *isd* locus (iron regulated surface determinant) was first found to be encoded on the genomes of *S. caprae* and *S. capitis*. These findings suggested that the plasmid and chromosome encoded *bla* operons of ECG species underwent different evolution paths, as well as they differed in the abundance of virulence genes associated with adherence, invasion, secretion system and immune evasion. Identification of *isd* loci in *S. caprae* and *S. capitis* indicated their ability to acquire heme as nutrient iron during infection.

## Introduction

Coagulase-negative *staphylococci* (CoNS) commonly live on the human skin (Piette and Verschraegen, 2009; Becker et al., 2014). They often caused infectious diseases in specific groups of patients, such as those with neonates, neutropenia, and so on (Ma et al., 2011; Zong et al., 2011), and infections at metastatic sites, such as joints, heart and bones. The infections in these populations are often not easy to treat (Casey et al., 2007). The virulence properties of CoNS species are mainly related to their ability to form biofilms and produce colonizing biomaterials (Becker et al., 2014). At present, these species are considered as important bloodstream pathogens usually with multidrug resistance (May et al., 2014). As a member of CoNS, *Staphylococcus caprae* commonly colonizes the milk gland and skin of goats and occasionally causes goat mastitis (Watanabe et al., 2018). In addition, *S. caprae* causes human infections, such as acute otitis externa (Shuttleworth et al., 1997), peritonitis (Shin et al., 2011), urinary tract diseases (Kanda et al., 1991), endocarditis (Vandenesch et al., 1995), meningitis (Benedetti et al., 2008), and many cases of bacteremia. However, the reasons why *S. caprae* can cause hospital-acquired infections haven't been fully elucidated.

Based on the 16S rRNA, *tuf* (elongation factor Tu), *rpoB* (β-subunit of RNA polymerase) and *dnaJ* (heat shock protein 40) genes (Lamers et al., 2012), Lamers et al. proposed a new classification and classified the *Staphylococcus* species into 15 cluster groups. This finding revealed that *S. caprae* was a member of“Epidermidis Cluster Group” (ECG) with *S. epidrmidis* as the leading causative organism which included *S. caprae, S. saccharolyticus, S. epidermidis, S. capitis* subsp. urealyticus and *S. capitis* subsp. capitis. The ECG species is composed of the “medium”-pathogenic staphylococci which means that when they are isolated from clinical specimens, it's uncertain that whether the infections are really caused by them (Becker et al., 2014). As *S. caprae, S. capitis* and *S. epidermidis* all belong to ECG, they are thought to share the basic mechanisms causing various hospital-acquired infections (Watanabe et al., 2018). Therefore, evaluating genomic structure divergences in virulence factors and metabolism is required to elucidate the mechanism of infections caused by ECG.

The complete set of conserved genes in all studied strains were defined as the core genome, while the accessory genome represents the genes existing in part of the strains, and the pan-genome comprises all genes in the core genome and accessory genome (Nourdin-Galindo et al., 2017; Wu et al., 2018). The pan-genome reflects the diversity among the species, host/environment adaptations as well as the variety of pathogenic mechanisms (Tettelin et al., 2008). In pan-genome analysis, unlike classical comparative genome analysis, all the genes were encompassed in a studied phylogenetic clade or a certain species (Chen et al., 2018) and will provide a better understanding of ECG genome diversity and virulence capabilities.

In this work, the whole genome sequence of a clinical *S. caprae* strain isolated from a puncture fluid specimen was determined. Furthermore, a genomic comparison among recently available ECGs was performed. Specifically, we analyzed the pan-genomes of each ECG species, as well as the virulence factors, and for the first time, we identified novel *isd* loci (iron uptake system allowing bacteria to steal iron from host heme) present in *S. caprae* and *S. capitis*. These genomic analyses will clarify the genomic differences, evolutionary relationships and pathogenic potentials of the strains.

## Materials and Methods

### Bacterial Strains, Genome Sequencing, Gene Predictions, and Functional Annotations

*S. caprae* SY333 was isolated from a puncture fluid specimen of a 29-year-old woman with fever in Lishui Hospital, Zhejiang, China. The strain was identified by Vitek-60 microorganism autoanalysis system (BioMerieux Corporate, Craponne, France), and then verified by homologous comparisons of 16S rRNA gene of *S. caprae* SY333 with those of the bacteria available in the nucleotide database of the National Center for Biotechnology Information (NCBI) (http://www.ncbi.nlm.nih.gov). Finally, the average nucleotide identity (ANI) was calculated to confirm the result.

The AxyPrep Bacterial Genomic DNA Miniprep kit (Axygen Scientific, Union City, CA, United States) was used to extract the genomic DNA of *S. caprae* SY333. Sequencing of the *S. caprae* SY333 genome was performed by a combination of technologies producing short (HiSeq 2500; Illumina) and long reads (MinION and PacBio RS II; Oxford Nanopore Technologies and Pacific Biosciences). Canu v1.8 (Koren et al., 2017) was used for initial assembly, and a hybrid assembly was subsequently performed using the Unicycler pipeline (Wick et al., 2017), with the contigs generated by Canu and all the sequenced reads (short and long reads) served as an input. The cyclization of final contigs was confirmed through the built-in tools of the unicycler. Other publicly available ECG genome sequences including 24 full genomes, and 44 draft genomes were downloaded from the NCBI public database (Table 1). Genes were predicted and annotated by using Prokka v1.14.0 (Seemann, 2014); furthermore, the predicted proteins were searched against the NCBI non-redundant (NCBI Resource Coordinators, 2016) and Swiss-Prot (UniProt Consortium, 2015) databases (Tatusov et al., 2003) using DIAMOND (Buchfink et al., 2015) with an e-value threshold of 1e-5. Annotation of the resistance genes was performed using ResFinder (Zankari et al., 2012) and Resistance Gene Identifier (RGI) software of Comprehensive Antibiotic Resistance Database version 4.0.3 (https://card.mcmaster.ca/) (McArthur et al., 2013) with an e-value threshold of 1e-10. CrisprCasFinder was employed to identify CRISPR/Cas system (Couvin et al., 2018).

**Table 1:** Genomes used for phylogenetic and pan-genome analyses in this study.

**Strains**	**Number of contigs**	**Number of proteins**	**Length (Mb)**	**GC content (%)**	**Accession number**	**Species**	**Genome state**	**Origin**
*Bacillus subtilis* 168	1	4,214	4.21	43.51	GCA_000009045.1	*B. subtilis*	Complete	NA
*S. capitis* 104_SEPI	43	2,444	2.56	32.86	GCA_001069155.1	*S. capitis*	Scaffold	Homo sapiens
*S. capitis* 129_SAUR	194	2,458	2.60	32.89	GCA_001060815.1	*S. capitis*	Scaffold	Homo sapiens
*S. capitis* 1341_SEPI	75	2,444	2.56	32.81	GCA_001069765.1	*S. capitis*	Scaffold	Homo sapiens
*S. capitis* 245_SAUR	120	2,406	2.54	32.81	GCA_001064095.1	*S. capitis*	Scaffold	Homo sapiens
*S. capitis* 441_SEPI	56	2,344	2.39	32.68	GCA_001071095.1	*S. capitis*	Scaffold	Homo sapiens
*S. capitis* 505_SAUR	52	2,462	2.58	32.85	GCA_001065245.1	*S. capitis*	Scaffold	Homo sapiens
*S. capitis* 562_SWAR	39	2,386	2.51	32.68	GCA_001073365.1	*S. capitis*	Scaffold	Homo sapiens
*S. capitis* 619_SEPI	57	2,429	2.54	32.82	GCA_001073565.1	*S. capitis*	Scaffold	Homo sapiens
*S. capitis* 622_SHAE	63	2,427	2.54	32.82	GCA_001073605.1	*S. capitis*	Scaffold	Homo sapiens
*S. capitis* 645_SEPI	40	2,437	2.55	32.87	GCA_001073715.1	*S. capitis*	Scaffold	Homo sapiens
*S. capitis* 658_SEPI	64	2,425	2.54	32.81	GCA_001073835.1	*S. capitis*	Scaffold	Homo sapiens
*S. capitis* 664.rep2_SAUR	40	2,448	2.56	32.85	GCA_001066795.1	*S. capitis*	Scaffold	Homo sapiens
*S. capitis* AYP1020	2	2,369	2.50	32.93	GCA_001028645.1	*S. capitis*	Complete	Homo sapiens (blood)
*S. capitis* C0756	27	2,380	2.50	32.79	GCA_003857115.1	*S. capitis*	Scaffold	Homo sapiens (Anterior Nose)
*S. capitis* C2784	34	2,396	2.50	32.73	GCA_003857145.1	*S. capitis*	Scaffold	Homo sapiens (Anterior Nose)
*S. capitis* C87	14	2,423	2.47	32.63	GCA_000183705.1	*S. capitis*	Scaffold	Homo sapiens (Upper respiratory tract)
*S. capitis* CR01	8	2,386	2.50	32.79	GCA_000499705.1	*S. capitis*	Scaffold	NA
*S. capitis* CR03	1	2,384	2.51	32.77	GCA_001215085.1	*S. capitis*	Scaffold	NA
*S. capitis* DSM 6717	184	2,338	2.47	32.90	GCA_002901925.1	*S. capitis*	Scaffold	Homo sapiens (skin)
*S. capitis* FDAARGOS_378	2	2,344	2.49	32.99	GCA_002591175.1	*S. capitis*	Complete	Homo sapiens (cerebrospinal fluid)
*S. capitis* NCTC 11045	51	2,366	2.43	32.67	GCA_002902325.1	*S. capitis*	Scaffold	Homo sapiens (skin)
*S. capitis* TW2795	2	2,360	2.49	33.05	GCA_002356175.1	*S. capitis*	Complete	Homo sapiens
*S. caprae* 26D	2	2,563	2.69	33.62	GCA_007814385.1	*S. caprae*	Complete	Buffalo milk
*S. caprae* 9557	85	2,627	2.75	33.34	GCA_000931485.1	*S. caprae*	Contig	Homo sapiens (cerebrospinal fluid)
*S. caprae* JMUB145	1	2,447	2.62	33.66	GCA_003966585.1	*S. caprae*	Complete	Homo sapiens (blood)
*S. caprae* JMUB590	1	2,466	2.63	33.61	GCA_003966605.1	*S. caprae*	Complete	Homo sapiens
*S. caprae* JMUB898	1	2,431	2.60	33.62	GCA_003966625.1	*S. caprae*	Complete	Homo sapiens
*S. caprae* M23864:W1	26	2,502	2.63	33.19	GCA_000160215.1	*S. caprae*	Scaffold	Homo sapiens (skin)
*S. caprae* NCTC 12196	101	2,468	2.61	33.51	GCA_002902725.1	*S. caprae*	Scaffold	Goat milk
*S. caprae* OG2-2	193	2,482	2.66	33.86	GCA_002276615.1	*S. caprae*	Contig	Kefir
*S. caprae* SNUC 4023	141	2,428	2.58	33.43	GCA_003578345.1	*S. caprae*	Contig	Bos taurus
*S. caprae* SY333	6	2,664	2.76	33.48	NA	*S. caprae*	Complete	Homo sapiens (puncture fluid)
*S. epidermidis* 1022_SEPI	137	2,447	2.60	31.91	GCA_001068475.1	*S. epidermidis*	Scaffold	Homo sapiens
*S. epidermidis* 1457	2	2,258	2.47	32.25	GCA_002085695.1	*S. epidermidis*	Complete	Homo sapiens (central venous catheter)
*S. epidermidis* 14.1.R1	4	2,523	2.63	32.18	GCA_001956655.2	*S. epidermidis*	Complete	Homo sapiens
*S. epidermidis* 949_S8	1	2,153	2.34	31.93	GCA_000934225.1	*S. epidermidis*	Chromosome	Homo sapiens
*S. epidermidis* ATCC 12228	7	2,350	2.56	32.05	GCA_000007645.1	*S. epidermidis*	Complete	NA
*S. epidermidis* ATCC 14990	3	2,259	2.49	32.22	GCA_006094375.1	*S. epidermidis*	Complete	NA
*S. epidermidis* BCM-HMP0060	46	2,394	2.61	31.27	GCA_000159575.1	*S. epidermidis*	Complete	Homo sapiens (skin)
*S. epidermidis* BPH0662	3	2,694	2.84	31.99	GCA_900086615.1	*S. epidermidis*	Complete	Homo sapiens
*S. epidermidis* BVS058A4	17	2,404	2.61	31.41	GCA_000314715.2	*S. epidermidis*	Scaffold	Homo sapiens
*S. epidermidis* CDC120	4	2,347	2.57	32.16	GCA_003856395.1	*S. epidermidis*	Complete	Homo sapiens (skin)
*S. epidermidis* CDC121	3	2,352	2.57	32.10	GCA_003856455.1	*S. epidermidis*	Complete	Homo sapiens (skin)
*S. epidermidis* CIM28	91	2,484	2.69	32.00	GCA_000418125.2	*S. epidermidis*	Scaffold	Mus musculus (skin)
*S. epidermidis* CSF41498	4	2,308	2.54	32.14	GCA_003325735.1	*S. epidermidis*	Complete	Homo sapiens (cerebrospinal fluid)
*S. epidermidis* DAR1907	1	2,574	2.73	32.09	GCA_002850315.1	*S. epidermidis*	Complete	Homo sapiens (blood)
*S. epidermidis* FDAARGOS 153	5	2,300	2.55	32.15	GCA_002944995.1	*S. epidermidis*	Complete	Homo sapiens (blood)
*S. epidermidis* FDAARGOS 161	3	2,276	2.52	32.15	GCA_002954055.1	*S. epidermidis*	Complete	Homo sapiens (blood)
*S. epidermidis* FDAARGOS 529	3	2,377	2.58	32.22	GCA_003812425.1	*S. epidermidis*	Complete	Homo sapiens (blood)
*S. epidermidis* HD33	3	2,315	2.49	32.03	GCA_006337225.1	*S. epidermidis*	Chromosome	Homo sapiens (skin)
*S. epidermidis* HD43	1	2,217	2.42	31.98	GCA_006337205.1	*S. epidermidis*	Chromosome	Homo sapiens (skin)
*S. epidermidis* HD66	3	2,341	2.52	32.00	GCA_006337185.1	*S. epidermidis*	Chromosome	Homo sapiens (skin)
*S. epidermidis* M0026	67	2,301	2.53	31.98	GCA_000551165.1	*S. epidermidis*	Scaffold	Homo sapiens (blood)
*S. epidermidis* M0881	9	2,443	2.68	31.57	GCA_000362145.1	*S. epidermidis*	Scaffold	Homo sapiens (blood)
*S. epidermidis* M23864:W2(gray)	13	2,324	2.52	31.61	GCA_000164075.1	*S. epidermidis*	Scaffold	Homo sapiens (skin)
*S. epidermidis* NBRC 100911	2	2,193	2.43	32.29	GCA_006742205.1	*S. epidermidis*	Complete	NA
*S. epidermidis* NCTC13924	1	2,643	2.75	31.99	GCA_900638695.1	*S. epidermidis*	Complete	Homo sapiens (blood)
*S. epidermidis* PM221	5	2,417	2.60	31.95	GCA_000751035.1	*S. epidermidis*	Complete	NA
*S. epidermidis* RP62A	2	2,434	2.64	32.15	GCA_000011925.1	*S. epidermidis*	Complete	Homo sapiens
*S. epidermidis* SE90	3	2,216	2.42	32.04	GCA_002749455.1	*S. epidermidis*	Chromosome	Homo sapiens (blood)
*S. epidermidis* SE95	5	2,220	2.44	31.94	GCA_002749515.1	*S. epidermidis*	Chromosome	Homo sapiens (blood)
*S. epidermidis* SEI	2	2,345	2.54	32.05	GCA_000759555.1	*S. epidermidis*	Complete	Homo sapiens
*S. epidermidis* Scl19	143	2,399	2.59	31.89	GCA_000418025.2	*S. epidermidis*	Scaffold	Mus spicilegus (skin)
*S. epidermidis* Scl22	490	2,119	2.37	32.18	GCA_000418045.1	*S. epidermidis*	Scaffold	Mus spicilegus
*S. epidermidis* Scl25	139	2,235	2.46	31.86	GCA_000418065.2	*S. epidermidis*	Scaffold	Mus spicilegus (skin)
*S. epidermidis* Scl31	401	2,275	2.53	31.90	GCA_000418085.2	*S. epidermidis*	Scaffold	Mus spicilegus (skin)
*S. epidermidis* W23144	91	2,403	2.65	31.06	GCA_000160235.1	*S. epidermidis*	Scaffold	Homo sapiens (skin)
*S. epidermidis* WI05	114	2,404	2.60	31.94	GCA_000418145.2	*S. epidermidis*	Scaffold	Mus musculus (skin)
*S. epidermidis* WI09	95	2,548	2.76	31.98	GCA_000418165.2	*S. epidermidis*	Scaffold	Mus musculus (skin)

### Antimicrobial Susceptibility Testing and Cloning Experiments

The minimum inhibitory concentration (MIC) was detected by agar dilution method recommended by Clinical and Laboratory Standards Institute (CLSI). The result was interpreted following the CLSI breakpoint criteria for *Staphylococcus* (CLSI, 2019). *Enterococcus faecalis* ATCC 29212 and *Escherichia coli* ATCC 25922 were used as reference strains for quality control. The resistance gene sequences (*aadD2* and *blaZ*) along with their promoter regions were PCR-amplified using the primers 5'-GCTCTAGAGCTTTCTATTATTGCAATGTGGAATTG-3' and 5'-CGGGATCCCGTCAAAATGGTATGCGTTTTGACACA-3' for aadD2, and 5'-CGGGATCCCGATTTAGCCATTTTGACACCTTCTTT-3' and 5'-CCAAGCTTGGTTAAAATTCCTTCATTACACTCTTGGCG-3' for *blaZ*, with each having a pair of flanking restriction endonuclease adapters (*Xbal*l and *Bam*HI for *aadD2*, and *Bam*HI and *Hin*dIII for *blaZ*). The PCR products were then eluted from agarose gel, digested with the corresponding restriction endonucleases, and ligated into the pAM401 and pUCP24 vectors, respectively. The recombinant plasmid (pAM401-*aadD2*) was transformed into *E. faecalis* JH2-2 via the calcium chloride method, and the transformants were cultured on brain heart infusion agar plates with chloramphenicol (16 μg/mL). The recombinant plasmid (pUCP24-*blaZ*) was extracted and further transformed into *E. coli* DH5α. The cloned resistance gene was verified by restriction endonuclease digestion and sequencing (Shanghai Sunny Biotechnology Co., Ltd., Shanghai, China). All plasmids and strains of this work were shown in Table 2.

**Table 2:** Bacteria and plasmids used in antimicrobial susceptibility testing and cloning experiments.

**Strains and plasmids**	**Relevant characteristic(s)**	**Reference or source**
**Strains**		
*_*	The wild strain of *staphylococcus caprae* SY333	This study
DH5α	*Escherichia coli* DH5α was used as a host for the cloned resistance genes	Our laboratory collection
JH2-2	*Enterococcus faecalis* JH2-2 used as the host for the resistance genes cloning and the recipient for the conjugation experiment; RIF^r^	Our laboratory collection
ATCC 29212	*Enterococcus faecalis* ATCC 29212 used as the quality control strain for the antimicrobial test	Our laboratory collection
ATCC 25922	*Escherichia coli* ATCC 25922 was used as the quality control for antimicrobial susceptibility testing	Our laboratory collection
DH5α/pUCP24	DH5α carrying vector pUCP24; GEN^r^	Our laboratory collection
JH2-2/pAM401	JH2-2 carrying vector pAM401, CHL^r^	Our laboratory collection
DH5α/pUCP24-ORF	DH5α carrying the recombinant plasmid pUCP24 cloned with resistance gene ORF with its upstream promoter region (*blaZ*)	This study
JH2-2/pAM401-ORFs	JH2-2 carrying the recombinant plasmids of pAM401 cloned with resistance genes ORF with its upstream promoter region (*aadD2*)	This study
**Plasmids**		
pUCP24	Cloning vector for the PCR products of resistance gene ORFs with the promoter regions; GEN^r^	Our laboratory collection
pAM401	Cloning vector for the PCR products of all resistance genes with the promoter regions; CHL^r^	Our laboratory collection

*ORFs, open reading frames; r, resistance; RIF, rifampin; GEN, gentamicin; CHL, chloramphenicol*.

### Phylogenetic and Phylogenomic Analyses

The ubiquitous, conserved single-copy genes, including *atpD, recA, gyrA, gyrB*, and *ftsZ* (Nourdin-Galindo et al., 2017; Chen et al., 2019), from each strain were used for phylogenetic analysis. *Bacillus subtilis* 168 was used as the outgroup. Initially, the nucleotide sequences of each gene was translated and then concatenated by a custom-derived shell script; Multi-FASTA alignment was performed using MAFFT v7.407 (Katoh and Standley, 2013), and the resulting alignment was used to infer the phylogeny by the maximum likelihood algorithm (ML) using RAxML version 8.2.12 (Stamatakis, 2014) under the substitution matrix LG, which was selected by ProtTest version 3.4 (Darriba et al., 2011).

For further verification, phylogenomic analysis (Comas et al., 2007) was conducted using 764 single-copy orthologous genes existing in all 69 ECG strains as well as *Bacillus subtilis* 168. These single-copy orthologous genes were identified using Orthofinder version 2.3.8 (Emms and Kelly, 2019). The methods of Multi-FASTA alignment and phylogeny inference were the same as those mentioned above.

### Pan-Genome Inference and COG Functional Characterization

The pan-genomes of each ECG species were inferred with Roary version 3.12.0 (Page et al., 2015). The annotation files generated by Prokka were provided to Roary as an input. The gene presence/absence matrix produced by Roary was listed in Supplementary Table 1, and further analysis was based on this file. The gene accumulation curve was produced via ggplot2 (Wickham, 2009) using the results of Roary. COG categorization of each pan-genome was carried out using DIAMOND, and reference gene sequences provided by Roary were searched against the COG database. Only those hits with an e-value <1e-10, an identity higher than 40% and a coverage higher than 70% were considered significant (Nourdin-Galindo et al., 2017).

### Identification of the Virulence Genes in ECG Genomes

BLASTX program was used to search all coding sequences of ECG strains against Virulence Factor Database (VFDB) (http://www.mgc.ac.cn/VFs/) (Chen et al., 2005). Compared with the virulent genes in the database at an e-value <1e-10, only those query genes with an identity higher than 40% and a coverage higher than 70% were considered as the potential virulence genes (Nourdin-Galindo et al., 2017). Functional annotations were performed based on the categories and subcategories presented in VFDB.

### Sequence Analysis of *bla* Region and *isd* Locus

Only the complete ECG genome sequences were selected to perform comparative genomic analyses of the *bla* region and *isd* locus. Nucleotide sequence alignment and construction of the neighbor-joining phylogenetic tree of the *bla* operon were performed using the MAFFT program and MEGAX (Kumar et al., 2018) with a bootstrap of 1,000 replicates. Since the *bla* regions belonging to the same structure are similar to each other, comparative genomics analysis was performed using several representative sequences of these three structures (**Figure 2**), and the results were visualized via the gggenes package in Rstudio. The near-iron transporter (NEAT) domains and secretion signal of *isd* proteins were predicted online using InterProScan software by searching against the InterPro database (Hunter et al., 2009). Typical features including a signal peptide (predicted using the SignalP server at www.cbs.dtu.dk/services/SignalP/) at the N terminus, the LPXTG-motif (identified using a custom-derived script written in Python) close to the C terminus followed by a hydrophobic transmembrane segment (predicted using the TMHMM server at www.cbs.dtu.dk/services/TMHMM-2.0/) and several positively charged residues at the C terminus (manually checked) of putative cell-wall-anchored proteins (four Isd proteins and OrfA) in *S. caprae* and *S. capitits* (Bowden et al., 2005) were predicted. Phylogenetic analysis of *isd* NEAT domains was carried out using the method described above. Comparisons of the *isd* loci were carried out using BLASTN and BLASTP. Other bioinformatics tools were written using Python and Biopython (Cock et al., 2009).

### Nucleotide Sequence Accession Number

The complete chromosome and five plasmids sequences of *S. caprae* SY333 (pSY333-92, pSY333-45, pSY333-41, pSY333-7, and pSY333-2) have been submitted to DDBJ/EMBL/GenBank under accession numbers CP051643, CP051644, CP051648, CP051645, CP051646, and CP051647, respectively.

## Results and Discussion

### General Features of the *S. caprae* SY333 Genome

The *S. caprae* SY333 genome consists of a circularly closed chromosome and five non-conjugative plasmids named as pSY333-92, pSY333-45, pSY333-41, pSY333-7, and pSY333-2. The chromosome of *S. caprae* SY333 is ~2.58 Mb in length with an average GC content of 33.72% and encodes 2,435 open reading frames (ORFs). These five plasmids (pSY333-92, pSY333-45, pSY333-41, pSY333-7, and pSY333-2) are all circular DNA sequences with 91,820, 44,854, 41,252, 7,385, and 1,983 bp in length, encoding 126, 56, 49, 9 and 1 ORFs, respectively. Staphylococcal plasmids range from just over 1.0 kb to >60.0 kb in size (Kwong et al., 2017), and the smaller plasmids (between 1.0 and 8.0 kb) generally replicate via a rolling-circle replication (RCR) mechanism that is hallmarked by the production of single-stranded intermediates during replication. The pSY333-2 harbors a single ORF which encodes a replication protein. Searching against NCBI nucleotide database showed that pSY333-2 shared a high sequence similarity (85% coverage and 83% identity) with a plasmid SAP108D (2,422 bp) from *S. epidermidis* and also encodes only one replication gene.

Up to date, there are only 10 genome sequences of *S. caprae* present in NCBI genome database, of which 6 are incomplete genome sequences. Among the 4 complete genome sequences, 3 (*S. caprae* JMUB145, JMUB590 and JMUB898) were isolated from human skin without any plasmid, and the other one (*S. caprae* 26D) was isolated from buffalo milk with a plasmid (carrying *blaZ*). The genome of *S. caprae* SY333 is the first one of a clinical *S. caprae* isolate carrying multiple plasmids, of which 2 are resistance plasmids.

### Resistance Genes and Their Functions in *S. caprae* SY333

A total of 5 drug resistance genes associated with 3 antibiotics classes (β-lactams, aminoglycosides, and macrolides) were identified in the *S. caprae* SY333 genome, with 2 on the chromosome (*norA* and *mgrA*), 2 on pSY333-45 (*aadD2* and *msrA*) and 1 on pSY333-41 (*blaZ*). Two resistance genes (*aadD2* and *blaZ*) were cloned for functional evaluation. Compared with the control strain (JH2-2/pAM401), the cloned *aadD2* gene increased 8- and >16-fold of the MIC levels to tobramycin and kanamycin, respectively. Meanwhile, the *blaZ* gene exhibited >512-, 128-, >256-, and >1024-fold increase in MIC levels in response to treatment with ampicillin, cephazolin, ceftazidime, and meropenem, respectively, compared with those of the control (DH5α/pUCP24) (Table 3). The *in vitro* susceptibility testing of *S. caprae* SY333 exhibited resistance to a number of antibiotics, including erythromycin and clarithromycin (macrolides), amikacin and azithromycin (aminoglycosides) and penicillin (β-lactam), according to CLSI breakpoint criteria for *Staphylococcus*. Moreover, the MIC level of roxithromycin against *S. caprae* SY333 was 128 μg/mL. Although there was no interpretation criteria of resistance breakpoint for roxithromycin, it was significantly higher than that for erythromycin (>8 μg/mL).

**Table 3:** MIC values of antibacterial drugs for all strains (μg/mL).

**Strains**	***S.caprae* SY333**	**ATCC 29212**	**ATCC 25922**	**JH2-2**	**JH2-2/pAM401**	**/JH2-2/pAM401-*aadD2***	**DH5α**	**DH5α/pUCP24**	**DH5α/pUCP24-*blaZ***
TOB	4	8	–	32	32	256	–	–	–
GEN	<0.25	4	–	8	8	8	–	–	–
KAN	8	32	–	64	64	>1024	–	–	–
STR	4	64	–	128	128	128	–	–	–
AMP	4	2	4	–	–	–	4	2	>1024
FOX	2	256	2	–	–	–	2	2	2
CZO	<1	16	2	–	–	–	<1	2	256
CAZ	4	512	0.25	–	–	–	0.25	<0.06	16
CTX	0.25	128	0.06	–	–	–	<0.06	<0.06	<0.06
MEM	0.125	4	0.06	–	–	–	<0.03	<0.03	>32
CIP	0.03	2	–	–	–	–	–	–	–
PEN	16	4	–	–	–	-	–	–	–
AMK	256	256	–	–	–	–	–	–	–
AZM	32	2	–	–	–	–	–	–	–
ERY	16	1	–	–	–	–	–	–	–
ROX	128	1	–	–	–	–	–	–	–
NAL	64	1024	–	–	–	–	–	–	–
CLR	16	<1	–	–	–	–	–	–	–

*TOB, Tobramycin; GEN, Gentamicin; KAN, Kanamycin; STR, Streptomycin; AMP, Ampicillin; FOX, Cefoxitin; CZO, Cephazolin; CAZ, Ceftazidime; CTX, Cefotaxime; MEM, Meropenem; CIP, Ciprofloxacin; PEN, Penicillin; AMK, Amikacin; AZM, Azithromycin; ERY, Erythromycin; ROX, Roxithromycin; NAL, Nalidixic acid; CLR, Clarithromycin. ATCC 29212, Enterococcus faecalis used as the quality control strain for the antimicrobial test; ATCC 25922, Escherichia coli used as the quality control for antimicrobial susceptibility testing; JH2-2, Enterococcus faecalis used as the host for cloned aadD2 gene; DH5α, Escherichia coli used as a host for the cloned blaZ gene*.

Two mechanisms have been reported to confer penicillin resistance in staphylococci (Olsen et al., 2006). The primary mechanism is the production of β-lactamase encoded by *blaZ*. The second is the expression of PBP2a, a penicillin-binding protein encoded by *mecA*. In this work, only one mechanism (*blaZ*) conferring penicillin resistance in the *S. caprae* SY333 genome was confirmed.

### Comparison of *bla* Region in ECG Species

**Figure 1:**
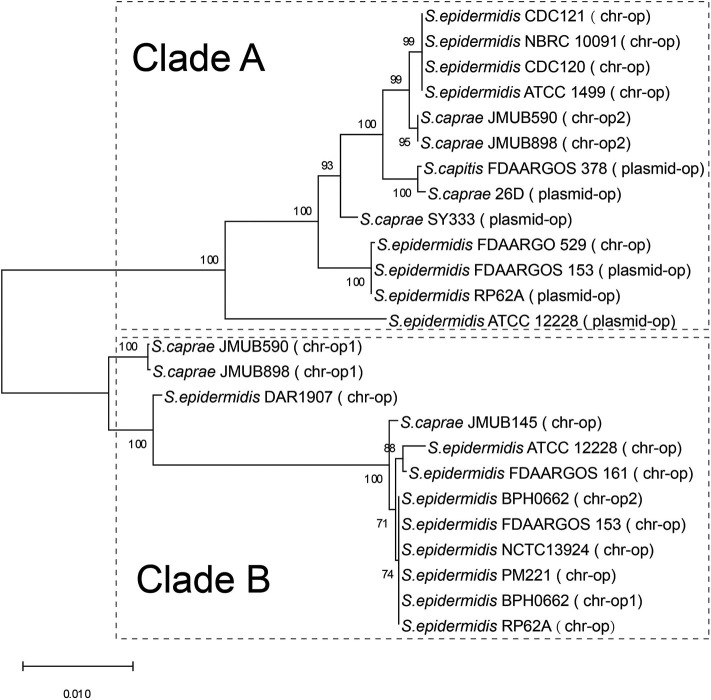
Phylogenetic relationships of *bla* operons among the complete genome sequences of ECG. The *bla* operon is abbreviated to op. chr-op1 indicates the first copy of the *bla* operon encoded on the chromosome, and chr-op2 indicates the second copy of the *bla* operon downstream of the first copy of the bacterium. Plasmid-op indicates the *bla* operon encoded on a plasmid.

The *bla* operon (*blaI*-*blaR1*-*blaZ*) carried by a Tn*552*-like element in *S. caprae* SY333 is encoded on the plasmid pSY333-41. According to serotyping and different hydrolyzing substrate profiles, the β-lactamases expressed by *blaZ* could be divided into four types A, B, C, and D (Pereira et al., 2014). Among them, type B is usually encoded in the chromosome, while the other three types (A, C, and D) are generally encoded on the plasmids (Bagcigil et al., 2012). Phylogenetic analysis of plasmid-borne and chromosomally located *bla* operons (*blaI*-*blaR1*-*blaZ*) in ECG species identified two distinct clades (clade A and clade B, Figure 1). Clade A included a mixture of strains with *bla* operons encoded on either chromosomes or plasmids, while Clade B only included strains in which the *bla* operon was encoded on the chromosome (Figure 1).

**Figure 2:**
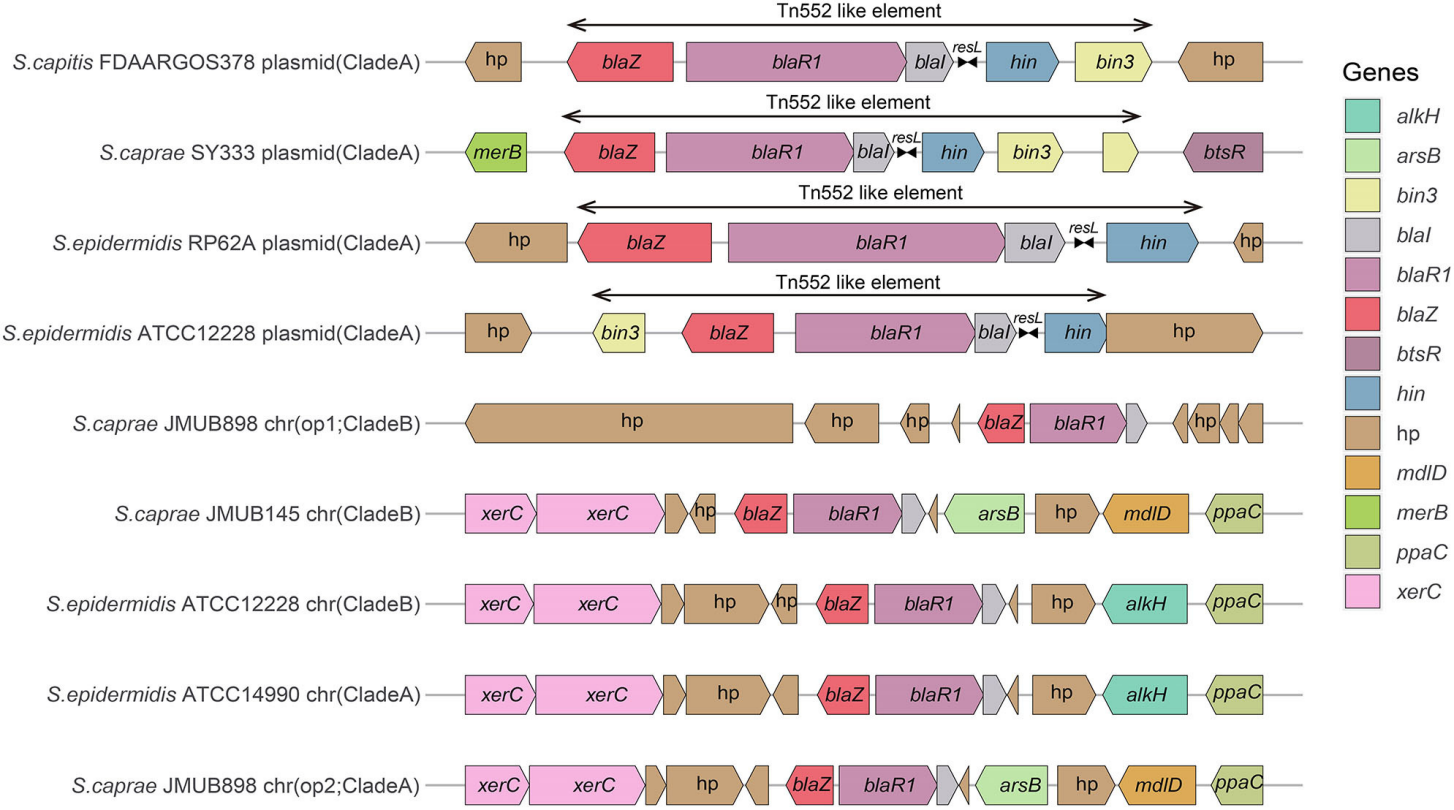
Comparison of the *bla* operons and their genetic environments. The genes are denoted by arrows and colored based on their assigned gene functions. The gene hp stands for hypothetical protein.

Comparison of the *bla* region (the *bla* operon and its immediate surroundings) showed that there were three distinct structures: (1) the *bla* operon was associated with a Tn*552*-like element that is commonly located on the plasmid, for example, *S. caprae* SY333 (plasmid-op) (except for *S. epidermidis* FDARGOS_529); (2) two tyrosine recombinase genes *xerC* were located downstream of the *blaZ* gene, which was only encoded on the chromosome, for example, *S. epidermidis* ATCC 12228 (chr-op); (3) the *bla* operon had no significantly featured surroundings and was only located on the chromosome, for example, *S. caprae* JMUB898 (chr-op1) (Figure 2). The β-lactamase gene-related transposon Tn*552* and its derivatives belong to a group of transposons targeting resolution sites (*resL*) and are almost ubiquitous in modern *S. aureus* isolates (Yui Eto et al., 2019). The above results indicated that the *bla* operon located on the chromosome and the plasmid had followed two distinct evolutionary paths, which has been confirmed by the hypothesis raised by Olsen et al. (2006). The appearance of the Tn*552*-like element in the chromosomes of *S. epidermidis* FDARGOS_529 also indicated that the *bla* operon-related region could be translocated by Tn*552* between chromosomes and plasmids. The fact that those chromosome-encoded *bla* regions clustered together with those plasmid-encoded regions indicated that they may originate from the same ancestors (Figure 1).

### Phylogenetic Relationship Among ECG Species

**Figure 3:**
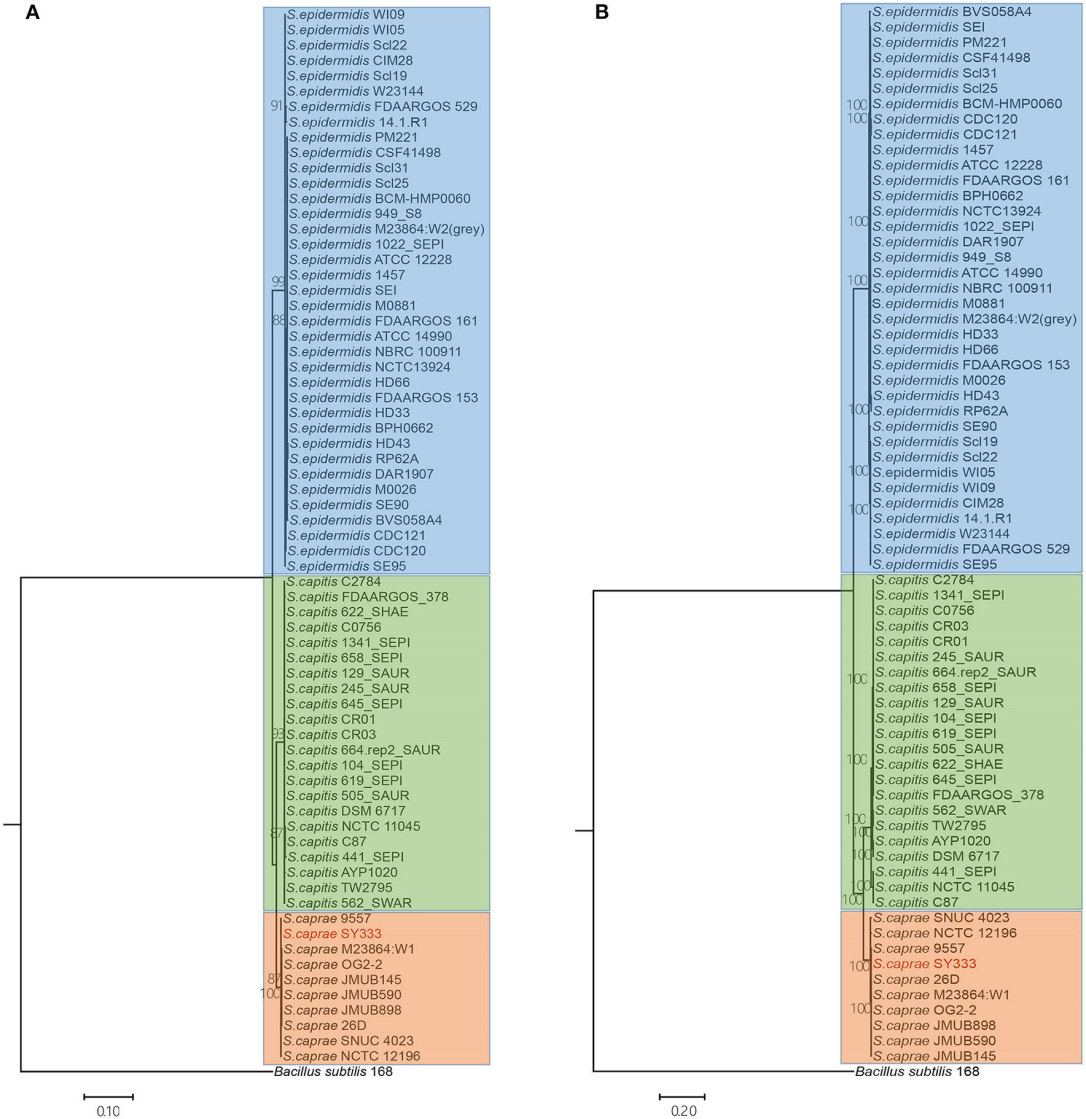
Phylogenetic and phylogenomics relationships of the housekeeping genes and single-copy orthologous genes of the ECG strains. **(A)** Phylogenetic relationship of ECG strains, according to the housekeeping genes shared by all complete genomes (i.e., *atpD, recA, gyrA, gyrB and ftsZ*). **(B)** Phylogenomics tree generated based on a total of 764 single-copy orthologous genes. Color shading indicates the species to which each stain belongs (blue: *S. epidermids*; green: *S. capitis*; orange: *S. caprae*). More information on strain characteristics is shown in Table 1.

To infer the phylogenetic relationship of the ECG strains, a phylogenetic tree using the five ubiquitously conserved core genes of the 70 strains (including *Bacillus subtilis*) was constructed. The resulting phylogenetic tree clearly grouped the 69 ECG strains into three distinct clades as expected. *S. caprae* was phylogenetically closer to *S. capitis* (Figure 3A). Through phylogenomics analyses of 764 concatenated genes, a tree similar to that reconstructed from five ubiquitously conserved core genes was obtained (Figure 3B). This inferred phylogenetic relationship among ECG species was highly consistent with a former report in which the phylogenetic relationship was reconstructed using single-nucleotide polymorphisms in 82 *Staphylococcus* genome sequences, including ECG species (Watanabe et al., 2018).

### Pan-Genome Inference and COG Functional Characterization

**Figure 4:**
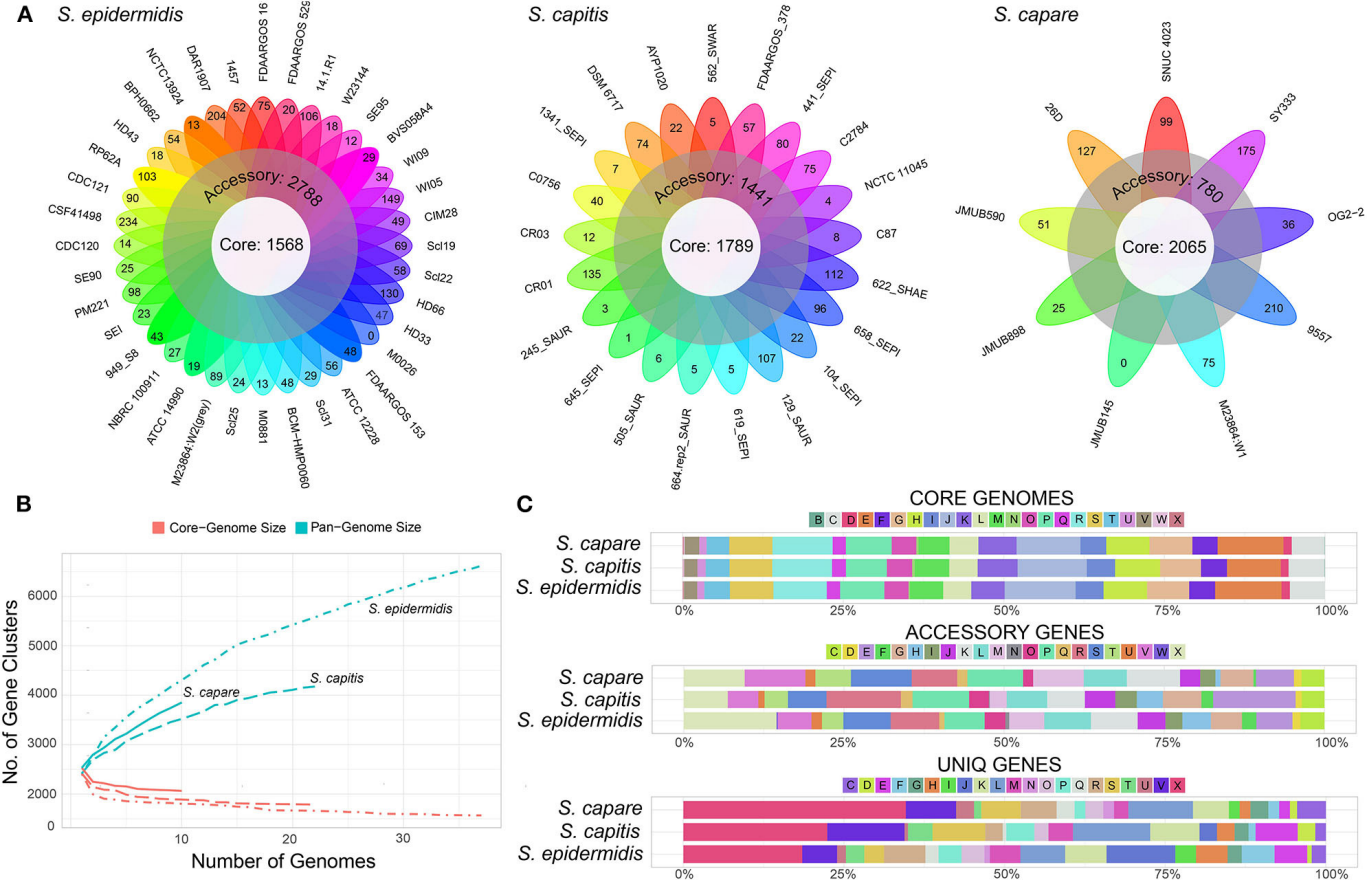
Representation of the pan-genomes and COG functional annotations of ECG species. **(A)** Comparative overview of ECG pan- and core-genomes. **(B)** Accumulation plots for ECG species pan- (blue) and core-genomes (red). The curves show the relationship among the pan-genomes, the core-genomes, and the number of genomes. As the number of genomes sequenced increased, pan-genomes also increased. **(C)** COG functional categories from the pan-genomes of ECG strains. COG categories are as follows: For cellular processes and signaling: D, cell cycle control, cell division and chromosome partitioning; M, cell wall/membrane/envelope biogenesis; N, cell motility; O, post-translational modification, protein turnover and chaperones; T, signal transduction mechanisms; U, intracellular trafficking, secretion and vesicular transport; V, defense mechanisms and Z, cytoskeleton. For information storage and processing: B, chromatin structure and dynamics; J, translation, ribosomal structure and biogenesis; K, transcription and L, replication, recombination and repair. For metabolism: C, energy production and conversion; E, amino acid transport and metabolism; F, nucleotide transport and metabolism; G, carbohydrate transport and metabolism; H, coenzyme transport and metabolism; I, lipid transport and metabolism; P, inorganic ion transport and metabolism and Q, secondary metabolite biosynthesis, transport and catabolism. X for mobilome or prophages. R for general function prediction only, and S for unknown function.

In order to compare the general genetic similarities and differences within the three species, the core and pan-genome of each species was determined. The result revealed that there were a total of 1,568 core genes, 2,788 accessory genes (genes of accessory genome present in at least two strains) and 2,263 unique genes (genes of accessory genome present in only one strain) among all *S. epidermidis* strains (Figure 4A). Similar to *S. epidermidis*, 1,789 core genes, 1,441 accessory genes, and 946 unique genes, and 2,065 core genes, 780 accessory genes and 1,008 unique genes were predicted among *S. capitis* strains and *S. caprae* strains, respectively.

The rare faction curve (Figure 4B) showed that as genomes were sampled, the genes never observed before are continuously added at a fairly steady rate, causing the pan-genome size to increase, with no sign of getting stable soon. This tended to indicate that the pan-genomes of the three species are “open” (Medini et al., 2005; Diene et al., 2013). In this work, the number and diversity of the *S. epidermidis* strains were greater than those of both *S. capitis* and *S. caprae*. This is the main reason why the pan-genome size of *S. epidermidis* is larger than the other two species (Figure 4A). To some extent, the pan-genome state (“open” or “close”) for an organism partially depended on its capacity of acquiring exogenous DNA (Diene et al., 2013), especially for the species living in bacterial communities, such as those skin inhabitants [coagulase-negative staphylococci (CoNS)]. These species had a high horizontal gene transfer range and were most likely to have an open pan-genome (Georgiades and Raoult, 2010). Moreover, several systems which prevent horizontal gene transfer (HGT) could also influence the pan-genome state, for example, the concomitant identification of CRISPR/Cas, RM and T/AT loci that constitute specialized systems preventing HGT in *S. lugdunensis* result in a closed pan-genome which was in contrast to all other staphylococci studied to date (Argemi et al., 2018). So far, however, these systems have rarely been identified in other CoNS (Argemi et al., 2018). In this study, only 2 (*S. capitis* CR01 and *S. capitis* CR03) and 3 strains (*S. epidermidis* FDAARGOS_153, *S. epidermidis* M0881 and *S. epidermidis* RP62A) of *S. capitis* and *S. epidermidis* were confirmed to harbor complete CRISPR/Cas system (Type III), while such system was more common in *S. lugdunensis*. To some extent, these factors led to open pan-genomes of ECG species analyzed in this work.

Functional classification according to COG category showed that, the core genes of the three species were successfully assigned to 23 subcategories which were more than those of the accessory genes and unique genes, suggesting that the core genes of the three species have been intensively studied. The core gene category repartition was highly similar among the 3 species, and was found to be abundant in “Translation, ribosomal structure and biogenesis,” “Amino acid transport and metabolism,” and “Energy production and conversion” (Figure 4C). However, the functions of accessory and unique genes of the three species showed significant diversity which could result in the diverse biological characteristics of ECG species. Compared to the core genomes, the accessory and unique gene pools of the ECG species contained significant abundance of the genes belonging to the class “Mobilome or prophages.” These genes often appear to transfer laterally between strains and result in the transmission of virulence and resistance genes between strains, thus influencing bacterial pathogenicity (Jackson et al., 2011).

### Virulence Genes in ECG Genomes

To analyze the different pathogenic potential within ECG, a total of 164 virulence genes from 69 ECG genomes were identified, and 57 (57/164, 34.8%) virulence genes coexisted in all ECG genomes. Interestingly, *S. caprae* carried the highest average number of virulence genes (128), and *S. caprae* SY333 carried the most virulence genes (134) among all 69 ECG genomes.

**Figure 5:**
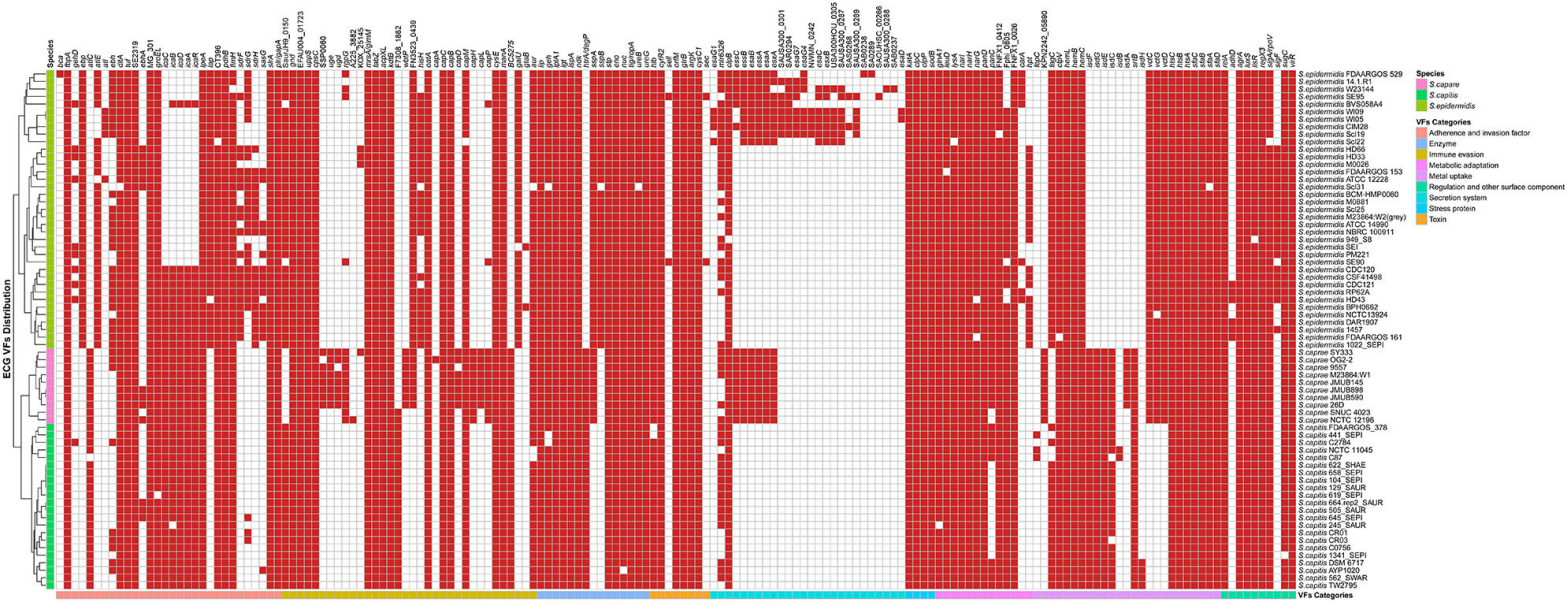
Distribution of virulence genes in ECG strains. The red square indicates the presence of the gene, while the white square indicates absence. The VF (virulence factor) categories and species are marked in the legend with different colors.

The virulence genes could be grouped into 8 categories according to the Virulence Factor Database, including “adherence and invasion factor,” “immune evasion,” “enzyme,” “toxin,” “stress protein,” “metabolic adaptation,” “metal uptake,” and “regulation and other surface component.” Remarkably, each species of ECG has its own specific virulence genes (Figure 5, Supplementary Figure 1). For *S. caprae*, the species-specific virulence genes were involved in “immune evasion” and “metal uptake”; for *S. capitis*, “metal uptake”; and for *S. epidermidis*, all categories except for “stress protein.” Most species-specific virulence genes in *S. caprae* belonged to “immune evasion,” especially those biosynthesis genes of capsular polysaccharide (*cap* locus), while in *S. epidermidis*, they mostly belonged to “secretion system.” In ECG genomes, most of the virulence genes were associated with adherence and immune evasion, indicating their strong ability to colonize their hosts and evade the innate immune system.

The genes involved in “enzyme,” “toxin,” “stress protein,” “metabolic adaptation,” and “regulation and other surface component” were relatively conserved in ECG genomes. Except for two toxin genes (*sell* and *sec*), which were only present in two strains of *S. epidermidis* (*S. epidermidis* SE90 and *S. epidermidis* SE95), the other toxin-related virulence factors were present in almost all strains of ECG. For secretion systems, the T6SS gene *clpB* and the T3SS gene *mlr6326* were commonly found in the ECG, while *EsaA, EssA, EssB*, and *EssC*, the proteins of T7SS machinery (Jäger et al., 2018), were only present in all strains of *S. caprae* and some of *S. epidermidis*. Notably, a secreted serine protease SspA (V8 protease) that degrades fibronectin-binding microbial surface components and recognizes adhesive matrix molecules (MSCRAMMs) to promote invasion (McGavin et al., 1997) was present in all strains of *S. caprae* and *S. epidermidis* but not *S. capitis*. In terms of intercellular aggregation, the *ica* (intercellular adhesion) locus involved in PNAG/PIA biosynthesis (Otto, 2009), seemed to be present more frequently in *S. capitis* and *S. caprae*, while those genes (*sdrFGH, ebp, ebh*, and *atlE*) associated with primary attachment appeared to be present almost exclusively in *S. epidermidis*. Previous studies have reported that most of the *S. epidermidis* strains carried SdrF, SdrG, and SdrH genes (Bateman et al., 2005). It has been reported that SdrG could necessarily and sufficiently promote *in vitro* adhesion to fibrinogen of *S. epidermidis* (Sun et al., 2005; Conrady et al., 2008) and *in vivo* central venous catheter (CVC) associated infectious disease (Guo et al., 2007). The enrichment of MSCRAMMs in *S. epidermidis* indicated that *S. epidermidis* achieved better *in vivo* colonization than the other two species. Although the *ica* locus is commonly present in *S. caprae* and *S. capitis*, the lack of MSCRAMM-related proteins may limit their ability to form biofilms (Otto, 2009). It is worth mentioning that *ica* and *cap* loci which protect the bacteria from the important innate host defense mechanisms are commonly present in *S. caprae* and might lead to better immune evasion (Otto, 2009; Fournier et al., 2013). T7SS, a secretion pathway for the virulence proteins, protects the bacteria from the host defense system and makes them able to survive in abscesses for a long time (Warne et al., 2016). Identification of T7SS in all *S. caprae* and some *S. epidermidis* strains indicated that these strains might possess higher capacity for secretion of virulence factors, as well as higher potential for bacterial pathogenesis. A previous study had shown that T7SS played an essential role in keeping integrity and homeostasis of the *staphylococcus aureus* membrane. This is crucial when the bacterium faces antimicrobial fatty acids (Lopez et al., 2017). Since T7SS is commonly present in *S. caprae*, it may suggest that T7SS targeted therapeutics decreases the virulence of *S. caprae* and makes it more susceptible to fatty acids. Similarly, the distribution of virulence factors involved in “enzyme,” “toxin,” “stress protein,” “metabolic adaptation,” and “regulation and other surface component” in ECG genomes indicates that ECG also has many similarities in terms of pathogenicity. Interestingly, in regard to “metabolic adaptation”-related genes, the *isd* genes have been only reported to be present in the species *S. lugdunensis* of CoNS (Heilbronner et al., 2011), while strains of *S. caprae* and *S. capitis* analyzed in this research also possess a gene cluster similar to those of *S. aureus* and *S. lugdunensis*.

### Comparative Genomics Analysis of *isd* Locus

In order to get iron from the host, pathogens have evolved several mechanisms. *S. aureus* uses the *isd* system as a fundamental heme-iron uptake pathway. The *S. aureus* genome encodes an *isd* system (*isdABCDEFGHI*) conferring heme uptake and sortase B (SrtB), which is responsible for anchoring its specific substrates (IsdC) to the bacterial cell wall (Grigg et al., 2010; Liang et al., 2016). The cell walls of *S. aureus* anchored four Isd proteins (IsdA, IsdB, IsdC, and IsdH). These proteins contain 1 to 3 conserved NEAT domains. Each Isd surface gene at least encodes a secretion signal, a cell wall-anchoring motif and a NEAT domain (Grigg et al., 2010; Heilbronner et al., 2016). IsdB (NEAT1) and IsdH (NEAT1 and NEAT2) can bind to hemoglobin and the haptoglobin-hemoglobin complex but not heme via N-terminal NEAT domains, while IsdH NEAT3 and IsdB NEAT2 contain heme-binding NEAT domains that transfer heme to IsdA, IsdC, and then to IsdEF, which is a membrane-locating transporter (Liu et al., 2008; Muryoi et al., 2008). IsdG and IsdI, the heme oxygenases in the cytoplasm, are responsible to destroy the porphyrin ring, releasing the free iron (Reniere et al., 2007).

**Figure 6:**
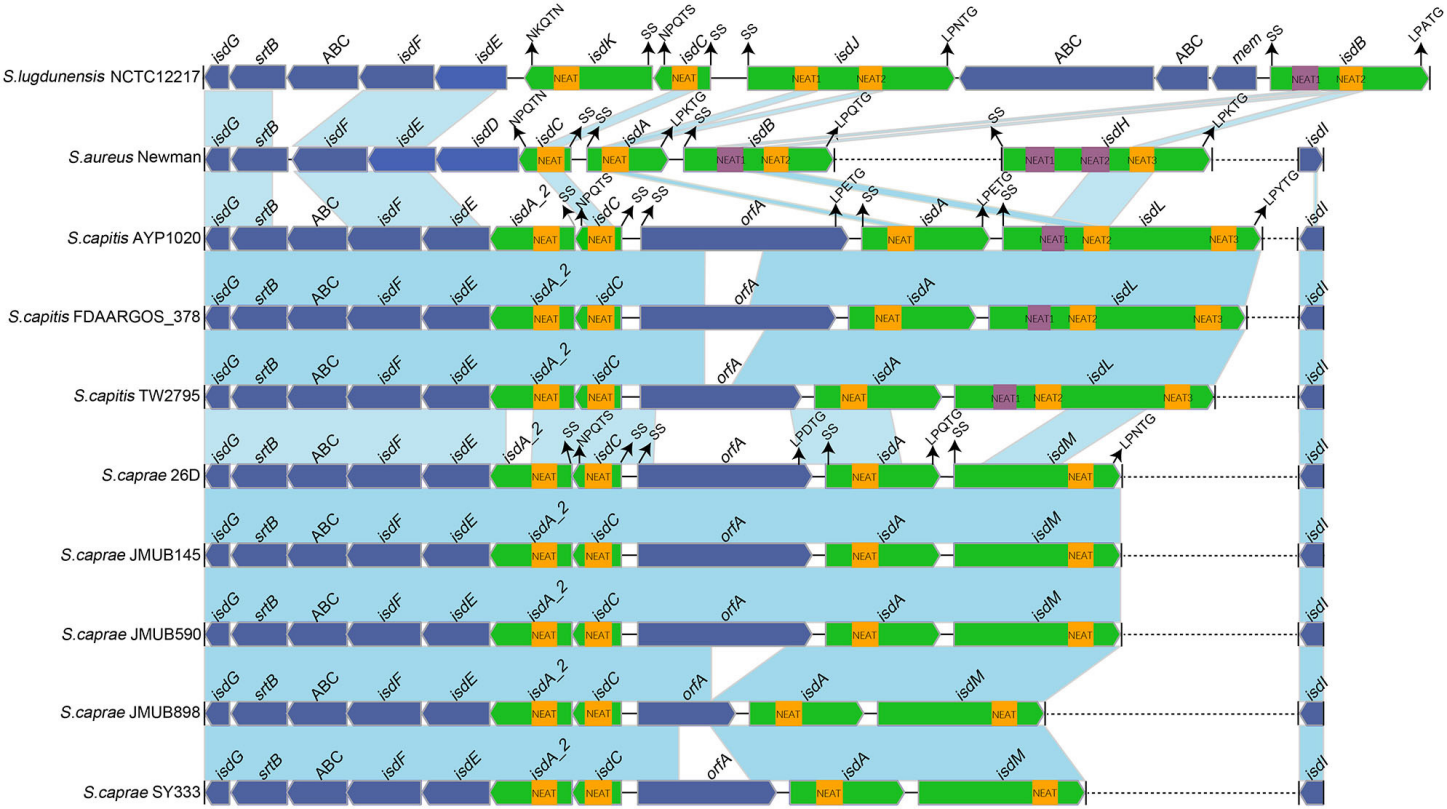
Comparison of the *isd* loci from completely sequenced genomes of *S. caprae* and *S. capitis* and the complete genomes of *S. aureus* Newman and *S. lugdunensis* NCTC12217. Blue links connect orthologous genes, and the color depth indicates the degree of identity. Heme-binding NEAT domains are indicated by yellow boxes, while Hb-binding NEAT domains are indicated by purple boxes. Genes are denoted by large arrows, and heme- or Hb-binding genes are colored in green. Dashed lines indicate genes that are not adjacent. The putative cell wall-anchoring motifs, single sequences and the function of NEAT domains in *S. aureus* Newman are indicated with arrows. SS, secretion signal; orfA, an unknown function putative cell wall-anchoring protein; ABC, ABC transporter.

**Figure 7:**
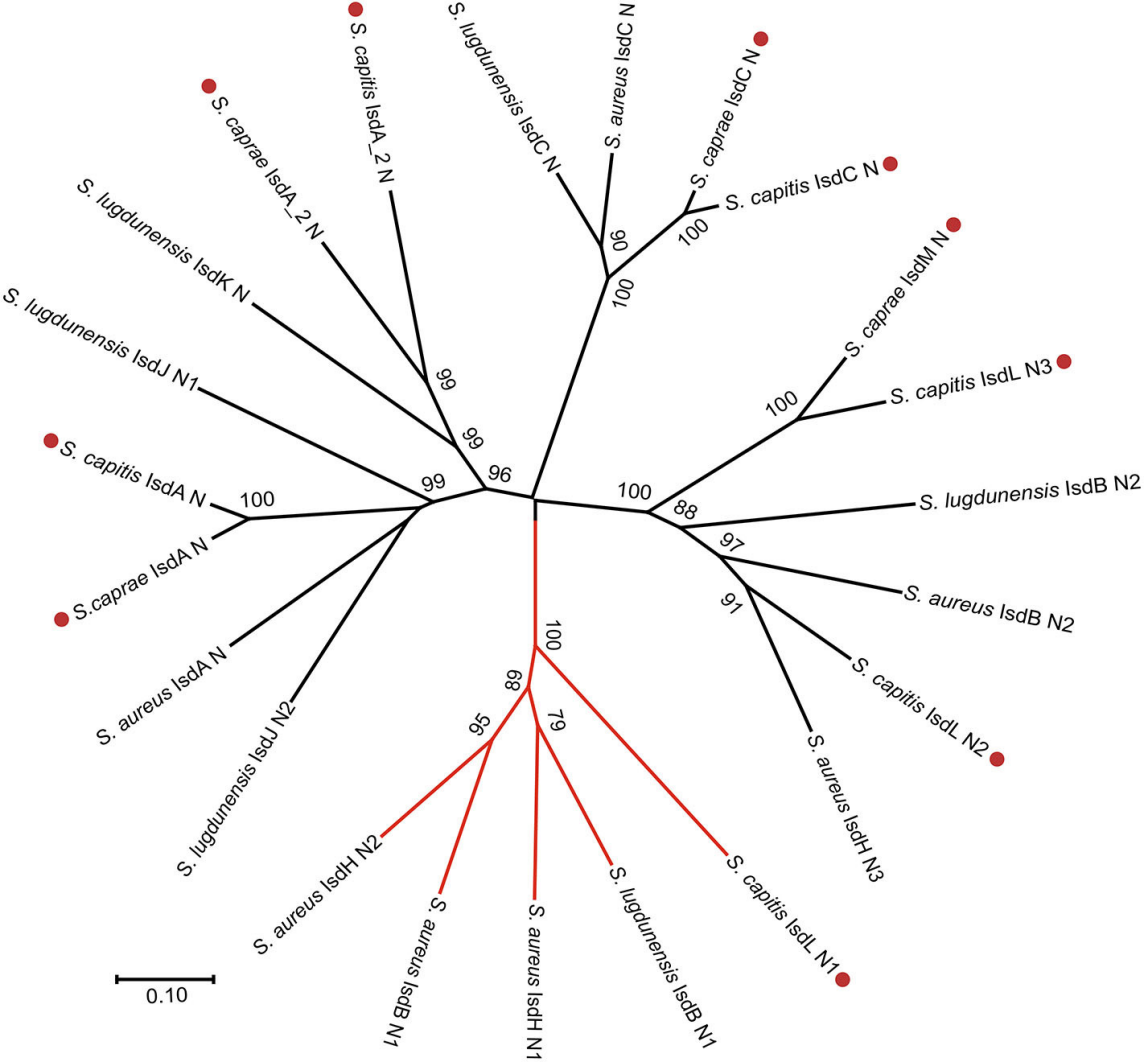
Phylogenetic relationships of NEAT domains in *S. capitis* AYP1020, *S. caprae* 26D, *S. lugdunensis* NCTC12217, and *S. aureus* Newman. Branches of Hb-binding NEAT domains are in red. Putative NEAT domains from *S. capitis* or *S. caprae* are followed by a red dot.

**Figure 8:**
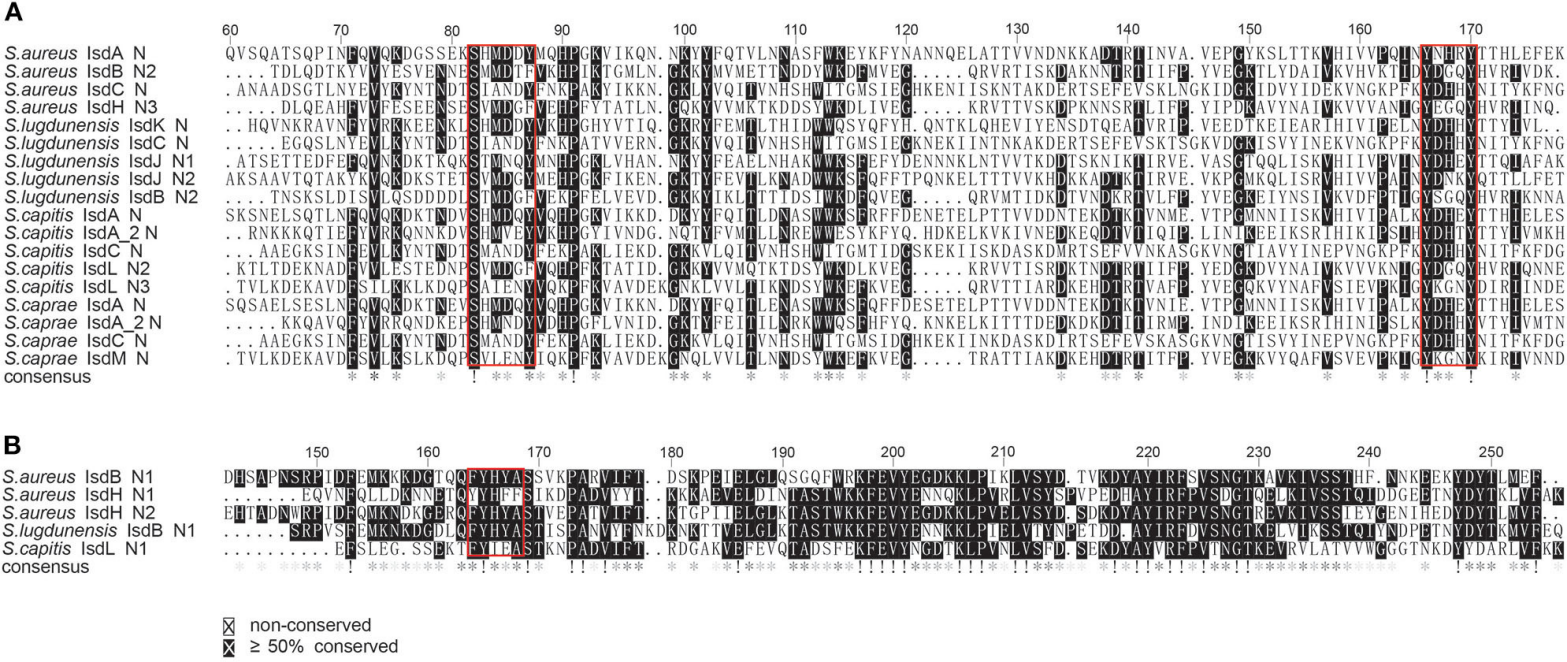
Multiple sequence alignments of the NEAT domains of *S. aureus* Newman, *S. lugdunensis* NCTC12217, *S. caprae* 26D and *S. capitis* AYP1020. **(A)** Alignment of the putative heme-binding NEAT domains of *S. caprae* 26D and *S. capitis* AYP1020. Conserved motifs are framed in red boxes. **(B)** Alignment of the putative Hb-binding NEAT domain of *S. capitis* AYP1020. Conserved motifs are framed in red box.

To better illustrate the similarities and differences of the *isd* locus among *S. capitis, S. caprae, S. aureus*, and *S. lugdunensis*, we performed a comprehensive comparison of the *isd* genes within these four species. There are four NEAT proteins in *S. caprae* (*isdA_2, isdC, isdA*, and *isdL*) and *S. capitis* (*isdA_2, isdC, isdA*, and *isdM*), respectively, which contain NEAT domains similar to those of *S. aureus* and *S. lugdunensis* (Table 4). Except for *isdA_2*, the other three NEAT-containing proteins in *S. capitis* and *S. caprae* possessed a secretion signal and a putative cell wall-anchoring motif (LPXTG) (Figure 6), which indicated the capacity of cell wall attachment (Cabanes et al., 2002). A comparative genome analysis demonstrated that the *isdGEFI* and *srtB* genes were conserved across *S. capitis, S. caprae*, and *S. aureus*, while other NEAT domain-containing genes appeared to be less conserved (Figure 6). IsdD, a membrane protein in *S. aureus*, is absent in *S. capitis* and *S. caprae* (Grigg et al., 2010). Phylogenetic analysis of NEAT domains showed that IsdL NEAT2 and NEAT3 and IsdM NEAT were phylogenetically close to IsdB NEAT2, IsdH NEAT3 of *S. aureus* and IsdB NEAT2 of *S. lugdunensis*, which were responsible for heme binding. IsdL NEAT1 was clustered together with IsdH NEAT1 and NEAT2, IsdB NEAT1 of *S. aureus* and IsdB NEAT1 of *S. lugdunensis* which were responsible for binding to the haptoglobin-hemoglobin complex and to hemoglobin (Figure 7). Multiple sequence alignments of NEAT domains revealed that each putative heme-binding NEAT domain in *S. capitis* and *S. caprae* contained an essential YXXXY heme-binding motif (Figure 8A). The first tyrosine of the heme-binding motif (e.g., Y166 of IsdA) non-covalently binds the central iron atom of heme, and the second tyrosine residue (e.g., Y170 of IsdA) provides a stabilizing hydrogen bond to the first tyrosine (Sheldon and Heinrichs, 2015). Except for IsdL NEAT2 of *S. capitis*, these putative heme-binding NEAT domains also contained a conserved SXXXXY sequence which forms a 3_10_-helix (α-helix) referred to as the “lip” region (Figure 8A). Hemoglobin binding by these NEAT domains in *S. aureus* is mediated by a conserved five amino acid aromatic motif (YYHFF in IsdH-N1 and FYHYA in IsdH-N2 and IsdB-N1 at positions 164 to 168). Mutation of any one of these key residues severely hinders hemoglobin and/or haptoglobin binding (Sheldon and Heinrichs, 2015). Alignment of the hemoglobin-binding NEAT domains revealed amino acid substitution at position 166 (H166I) and 167 (Y167E) in IsdL NEAT1 which might confer reduction or loss of the function (Figure 8B). These results indicate that these putative heme-binding NEAT domains in *S. capitis* and *S. caprae* have the ability to bind to heme, while whether the putative hemoglobin-binding domain (IsdL NEAT1) in *S. capitis* is functional needs further validation.

**Table 4:** Identities and similarities among NEAT domains of *S. aureus* Newman, *S. lugdunensis* NCTC12217, *S. caprae* 26D, and *S. capitis* AYP1020.

	***S.caprae*** **26D**				***S.capitis*** **AYP1020**					
**% identities/ % similarities**	**IsdA NEAT**	**IsdA_2 NEAT**	**IsdC NEAT**	**IsdM NEAT**	**IsdA NEAT**	**IsdA_2 NEAT**	**IsdC NEAT**	**IsdL NEAT1**	**IsdL NEAT2**	**IsdL NEAT3**
IsdA NEAT_SA_	56.03/77.59	32.41/59.26	25.83/47.50	32.11/47.71	56.03/78.45	37.04/64.81	25.00/46.67	-	28.30/44.34	30.91/50.00
IsdB NEAT1_SA_	-	27.85/49.37	–	–	28.30/49.06	23.81/50.48	–	45.26/65.26	–	–
IsdB NEAT2_SA_	29.59/44.90	23.15/44.44	26.04/47.92	43.75/64.29	28.57/45.92	–	27.08/47.92	–	63.21/78.30	41.07/65.18
IsdC NEAT_SA_	33.04/46.09	27.93/47.75	72.65/88.03	26.32/47.37	34.23/46.85	–	70.09/86.32	–	–	–
IsdH NEAT1_SA_	–	–	–	–	–	–	–	40.95/68.57	–	–
IsdH NEAT2_SA_	24.71/47.06	25.00/42.50	–	–	24.14/44.83	31.34/56.72	–	52.58/76.29	–	–
IsdH NEAT3_SA_	–	19.42/43.69	–	41.67/63.89	–	–	–	–	70.09/83.18	41.67/65.74
IsdJ NEAT1_SL_	50.43/70.43	37.96/59.26	30.36/39.29		49.57/69.57	34.26/61.11	30.36/42.86	–	–	–
IsdJ NEAT2_SL_	52.10/74.79	39.09/62.73	21.49/43.80	28.30/46.23	51.26/73.11	36.36/65.45	20.66/42.98	–	29.63/47.22	27.36/48.11
IsdKNEAT_SL_	42.73/65.45	50.00/70.00	22.94/42.20	23.42/52.25	41.82/64.55	44.55/70.00	22.94/42.20	23.75/47.50	23.81/42.86	23.42/49.55
IsdCNEAT_SL_	28.83/47.75	31.53/49.55	77.39/90.43	28.38/50.00	29.73/48.65	–	73.04/89.57	–	25.71/47.14	30.67/50.67
IsdBNEAT1_SL_	29.41/50.00	30.99/47.89	–	–	30.88/47.06	29.11/51.90	–	40.82/65.31	–	–
IsdBNEAT2_SL_	28.18/49.09	26.21/49.51	32.88/54.79	47.66/68.22	26.36/47.27	21.36/45.63	32.88/53.42	–	55.96/75.23	46.79/69.72

*Table comparison of the amino acid conservation between NEAT domains of S. aureus Newman and S. lugdunensis NCTC12217 (vertical) and S. caprae 26D and S. capitis AYP1020 Isd proteins (horizontal) as indicated. –, no obvious homology between the domain's amino acid chains. Identity defines the percentage of amino acids (or nucleotides) with a direct match in the alignment. SA indicating domains from S. aureus Newman and SL indicating domains from S. lugdunensis NCTC12217. The percent similarity of two sequences is the sum of both identical and similar matches (residues that have undergone conservative substitution)*.

In this study, we haven't found any NEAT domain that might bind to hemoglobin in *S. caprae*, and the function of the putative hemoglobin-binding domain (IsdL NEAT1) is still questionable. Indeed, not all heme or hemoglobin-binding Isd proteins contain the LPXTG motif, for example, *isdX1* (containing one NEAT domain) and *isdX2* (containing five NEAT domains) which are located between *isdC* and *isdE* in the *isd* loci of *Bacillus cereus* group were secreted without a LPXTG motif (Sheldon and Heinrichs, 2015). Unlike *S. aureus*, whose NEAT proteins acquire heme from hemoglobin directly at the bacterial surface, *Bacillus cereus* group secretes IsdX1 to capture heme in the extracellular milieu and relies on NEAT-NEAT interactions to deliver the bound heme to the envelope via IsdC (Maresso et al., 2008). Of note, *isdA_2* in *S. caprae* and *S. capitis* which is located between *isdC* and *isdE* also lacks LPXTG motif and that is similar to *isdX1* and *isdX2* although no obvious sequence identities were observed between *isdA_2* and *isdX* (*isdX1* and *isdX2*). Based on the characteristics of *isdA_2*, we hypothesized that IsdA_2 could be secreted out of bacteria cell rather than attached to the cell wall and interact with heme in the extracellular milieu. In addition, a putative cell wall-anchored protein OrfA was found in the *isd* loci of *S. capitis* and *S. caprae*, the function of OrfA remains unknown. In summary, the operation mechanism of Isd systems in *S. caprae* and *S. capitis* might be slightly different from that of *S. aureus* and *S. lugdunensis*.

*S. lugdunensis* was once considered as a unique species that harbors an iron-regulated surface determinant locus (*isd*) among coagulase-negative staphylococci. Discovery of the *isd* locus in *S. capitis* and *S. caprae* would provide evidence for their ability to use heme as an iron source during infection.

## Conclusion

In this work, the complete genome sequence of a clinical *S. caprae* isolate with two resistance plasmids was reported for the first time. *S. caprae* SY333 showed resistance to several antibiotics, such as erythromycin, clarithromycin, amikacin, azithromycin and penicillin. Two plasmid-encoded resistance genes (*blaZ* and *aadD2*) were confirmed to be functional. The pan-genome analysis of the three ECG species showed that their pan-genomes tend to be “open” and functional annotation revealed that core gene category repartition was highly similar across the 3 species. Analysis of the *bla* region in ECG revealed that the chromosome-encoded and plasmid-encoded *bla* operons had two distinct evolutionary paths. Virulence factors in ECG differed mostly in adherence, invasion, immune evasion and secretion system. T7SS may play an important role in pathogenesis of *S. caprae* and *S. epidermidis*. Genes related to primary attachment are almost exclusively present in *S. epidermidis*, while intercellular adhesion-related genes are more frequently present in *S. caprae* and *S. capitis*. Identification of the *isd* locus in *S. caprae* and *S. capitis* discouraged previous claims that *S. lugdunensis* was the only coagulase-negative Staphylococcus species with a locus encoding iron-regulated surface determinant (Isd) proteins, as well as indicated that this two species may have the ability to use heme as the nutrient iron source during infection, which could enhance their pathogenic potential.

## Data Availability Statement

This article contains previously unpublished data. The name of the repository and accession number(s) are not available.

## Ethics Statement

This study was approved by the Ethics Committee of the Central Hospital of Lishui City (China) and informed consent was obtained from the patient.

## Author Contributions

ZS, DZ, XZ, QL, HLin, WL, HLiu, and JL collected the strains and performed the experiments. ZS, XL, TX, and HZ analyzed the experimental results and performed the bioinformatics analysis. ZS, TX, QB, and HZ wrote the manuscript. KL, TX, QB, and HZ designed the experiments. All authors read and approved the final manuscript.

## Conflict of Interest

The authors declare that the research was conducted in the absence of any commercial or financial relationships that could be construed as a potential conflict of interest.

## Supplementary Material

Supplementary Table 1 Gene presence/absence matrix produced by Roary.

Supplementary Figure 1 A network diagram of all virulence factors (VFs) identified in ECG genomes, “Shared” means VFs present in three species, “Accessory” means VFs present in two species and “Species-specific” means VFs present in one species only.
