# Supplementary figures and images for: Determining the Genetic Characteristics of Resistance and Virulence of the “Epidermidis Cluster Group” Through Pan-Genome Analysis
Source: Front Cell Infect Microbiol. 2020 Jun 12;10:274. doi: 10.3389/fcimb.2020.00274 (PMC7303328; doi:10.3389/fcimb.2020.00274)

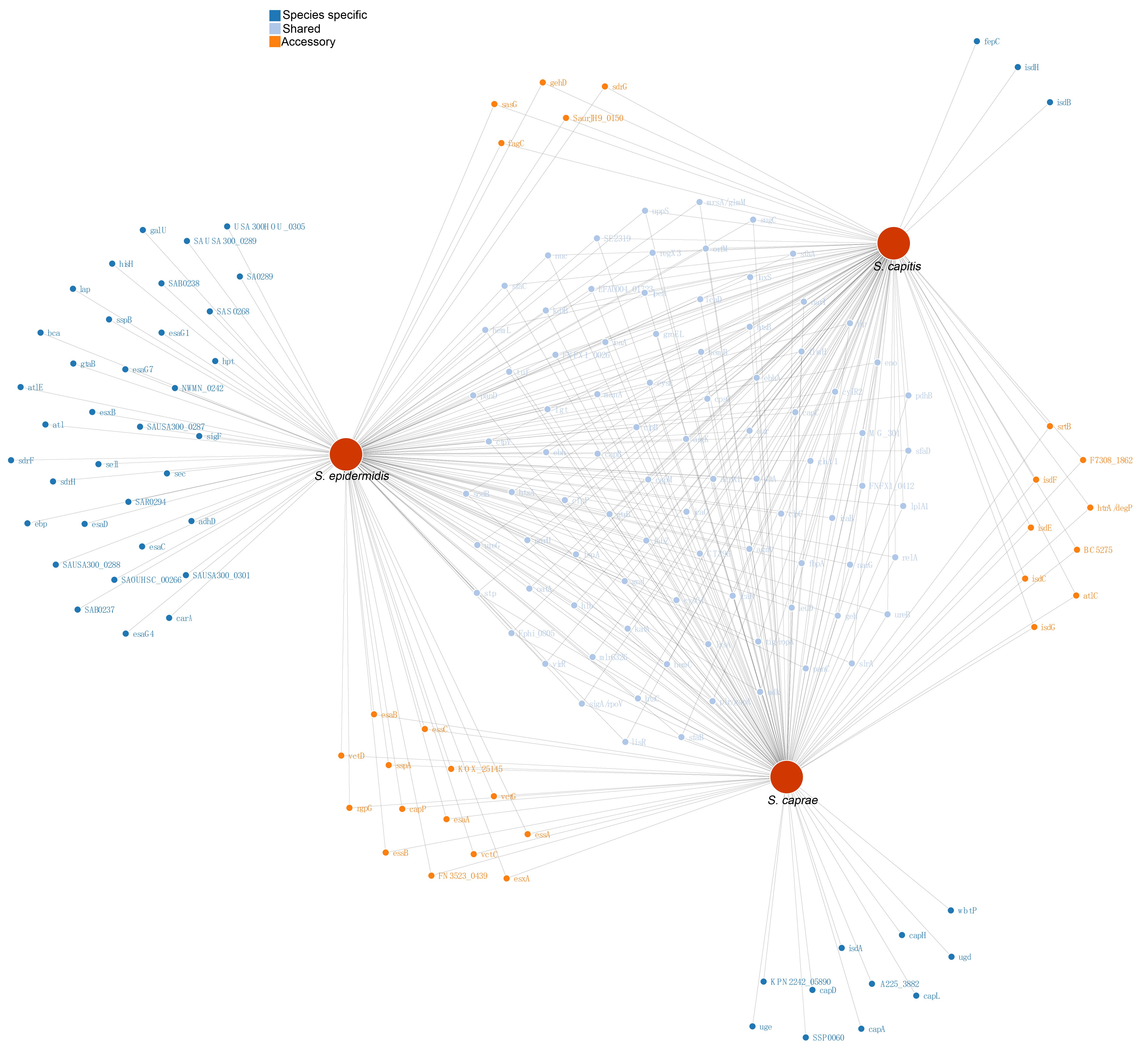

Supplement: Supplementary Figure 1 — A network diagram of all virulence factors (VFs) identified in ECG genomes, “Shared” means VFs present in three species, “Accessory” means VFs present in two species and “Species-specific” means VFs present in one species only. [file Image_1.JPEG]
